# Dietary Intake of Magnesium or Calcium and Chemotherapy-Induced Peripheral Neuropathy in Colorectal Cancer Patients

**DOI:** 10.3390/nu10040398

**Published:** 2018-03-23

**Authors:** Evertine Wesselink, Renate M. Winkels, Harm van Baar, Anne J. M. R. Geijsen, Moniek van Zutphen, Henk K. van Halteren, Bibi M. E. Hansson, Sandra A. Radema, Johannes H. W. de Wilt, Ellen Kampman, Dieuwertje E. G. Kok

**Affiliations:** 1 Division of Human Nutrition, Wageningen University & Research, Stippeneng 4, 6708 WE Wageningen, The Netherlands; vera.wesselink@wur.nl (E.W.); renate.winkels@wur.nl (R.M.W.); harm.vanbaar@wur.nl (H.v.B.); anne.geijsen@wur.nl (A.J.M.R.G.); moniek.vanzutphen@wur.nl (M.v.Z.); ellen.kampman@wur.nl (E.K.); 2 Department of Internal Medicine, Admiraal de Ruyter Ziekenhuis, ‘s-Gravenpolderseweg 114, 4462 RA Goes, The Netherlands; h.vanhalteren@adrz.nl; 3 Department of Surgery, Canisius Wilhelmina Ziekenhuis, Weg door het Jonkerbos 100, 6532 SZ Nijmegen, The Netherlands; b.hansson@cwz.nl; 4 Department of Medical Oncology, Radboud University Medical Centre, Geert Grooteplein-Zuid 22, 6525 GA Nijmegen, The Netherlands; sandra.radema@radboudumc.nl; 5 Department of Surgery, Radboud University Medical Centre, Geert Grooteplein-Zuid 22, 6525 GA Nijmegen, The Netherlands; Hans.deWilt@radboudumc.nl

**Keywords:** calcium, chemotherapy, colorectal cancer, magnesium, neuropathy, oxaliplatin

## Abstract

Chemotherapy-induced peripheral neuropathy (CIPN) is a common and severe side-effect in colorectal cancer (CRC) patients. This study assessed the association between habitual dietary intake of magnesium or calcium and prevalence and severity of chronic CIPN in CRC patients receiving adjuvant chemotherapy. For this prospective cohort study, 196 CRC patients were considered. Magnesium and calcium intake was determined using a food frequency questionnaire at diagnosis, during and after chemotherapy. Chronic CIPN was assessed 12 months after diagnosis using the quality of life questionnaire CIPN20. Prevalence ratios were calculated to assess the association between magnesium or calcium intake and the prevalence of CIPN. Multivariable linear regression analysis was used to assess the association between magnesium or calcium intake and severity of CIPN. CIPN was reported by 160 (82%) patients. Magnesium intake during chemotherapy was statistically significantly associated with lower prevalence of CIPN (prevalence ratio (PR) 0.53, 95% confidence interval (CI) 0.32, 0.92). Furthermore, higher dietary intake of magnesium during (β −1.08, 95% CI −1.95, −0.22) and after chemotherapy (β −0.93, 95% CI −1.81, −0.06) was associated with less severe CIPN. No associations were found for calcium intake and the prevalence and severity of CIPN. To conclude, we observed an association between higher dietary magnesium intake and lower prevalence and severity of CIPN in CRC patients.

## 1. Introduction

Chemotherapy-induced peripheral neuropathy (CIPN) is a common side-effect in colorectal cancer (CRC) patients treated with oxaliplatin [1]. Oxaliplatin can cause both acute and chronic CIPN. Symptoms of chronic CIPN include distal paresthesia, tingling sensations and numbness. Chronic CIPN predominantly affects sensory nerves and can lead to long-term disability [1]. Occurrence and severity of chronic CIPN are related to the cumulative dose and dose-intensity of the treatment [1,2,3]. Six to eight months after the end of oxaliplatin treatment, 40–60% of the patients still suffer from CIPN [4,5,6,7]. In order to minimize the detrimental effects in CRC patients, it is important to understand which factors can influence the development and severity of CIPN.

Magnesium and calcium are proposed to be important in the etiology of CIPN, because they are both involved in electric excitability of neurons and muscle contraction [8]. Low circulating levels of magnesium, but not calcium, before treatment have been associated with more severe CIPN in CRC patients [3]. Besides circulating levels of magnesium and calcium, several studies focused on intravenous administration of magnesium and calcium and the severity of CIPN [9,10,11,12,13,14,15,16]. The clinical observational study of Gamelin et al. found a lower occurrence and severity of CIPN in CRC patients who received magnesium and calcium in comparison to patients who did not receive magnesium and calcium for several reasons [9]. Other observational studies in CRC patients did not find an association between intravenous magnesium and calcium infusions and CIPN [10,11].

Several randomized-controlled trials (RCTs) [11,12,13,14,15] studied the effect of intravenous magnesium and calcium infusions on CIPN, but results are inconclusive. Grothey et al. reported a protective effect of magnesium and calcium on the prevalence of CIPN in 102 colon cancer patients [12]. The prevalence of CIPN was 22% in the magnesium and calcium group compared to 41% in the placebo group (*p* = 0.04) [12]. On the contrary, four other RCTs conducted among 27–353 CRC patients, did not find a protective effect of magnesium and calcium infusions on CIPN [13,14,15,16]. So far, no consensus about the use of magnesium and calcium infusions to reduce or prevent CIPN has been reached [17].

Most studies conducted so far focused on acute CIPN during and directly after treatment. It has been shown that further progression of CIPN can occur months after the end of treatment [1]. Moreover, the associations between dietary magnesium or calcium intake and the prevalence and severity of chronic CIPN have not been described so far. Dietary intake in relation to CIPN is important as dietary magnesium intake is the main contributor to the magnesium status. Further insights into the relation between diet and CIPN may provide feasible opportunities for dietary strategies directed against CIPN in cancer patients. In this prospective cohort study, we assessed the association between habitual dietary intake of magnesium or calcium and the prevalence and severity of CIPN approximately six months after chemotherapy, i.e., 12 months after diagnosis, in CRC patients receiving adjuvant chemotherapy.

## 2. Materials and Methods

### 2.1. Patients

**Figure 1:**
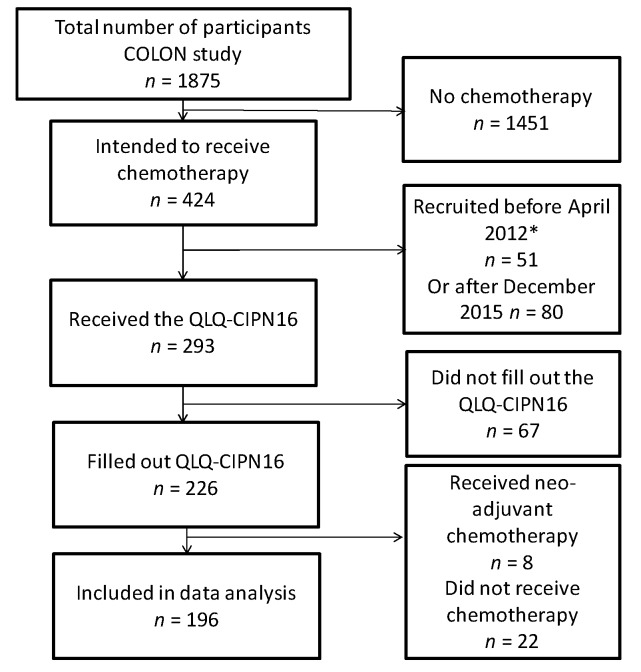
Flowchart representing patient selection for the current study. Colorectal cancer patients participating in the longitudinal observational study on nutritional and lifestyle factors (the COLON study) and who received adjuvant chemotherapy and filled out the quality of life questionnaire to assess chemotherapy-induced peripheral neuropathy (QLQ-CIPN16) were included in the present study. * Patients who were recruited before April 2012 were not included in this study, because the QLQ-CIPN16 questionnaire was implemented from April 2012 onwards.

This study is embedded in the longitudinal observational study on nutritional and lifestyle factors (COLON study), which is a prospective cohort study focusing on nutritional and lifestyle factors in relation to clinical outcomes and quality of life of CRC patients in the Netherlands [18]. The design and recruitment of the COLON study has been described in detail previously [18]. In short, newly diagnosed CRC patients were recruited directly after diagnosis in 12 hospitals in the Netherlands and were followed up during and after treatment. Men and women of all ages, who were in any stage of the disease were eligible for the study. Non-Dutch speaking patients, or patients with a history of CRC or (partial) bowel resection, chronic inflammatory bowel disease, hereditary CRC syndromes (e.g., Lynch syndrome, Familial Adenomatous Polyposis, Peutz-Jegher), dementia or another mental condition that made it impossible to fill out a questionnaire correctly, were excluded from the study. Participants of the COLON study who were included between April 2012 and December 2015 and who received adjuvant chemotherapy were eligible for the current study (*n* = 293). Patients who also received neoadjuvant chemotherapy (*n* = 8), who did not start with chemotherapy (*n* = 22), or did not fill out the questionnaire on CIPN (*n* = 67) were excluded, resulting in a total of 196 newly diagnosed CRC patients eligible for the current study (Figure 1). Hospital records and linkage with the Dutch ColoRectal Audit (DCRA) [19] were used to obtain data regarding cancer type, cancer stage and type of chemotherapy. All patients provided written informed consent. All procedures followed were in accordance with the ethical standards of the institution and the COLON study was approved by the Committee on Research involving Human Subjects, region Arnhem-Nijmegen, the Netherlands (file 2009-349).

### 2.2. Magnesium and Calcium Intake

A 204-item semi-quantitative food frequency questionnaire (FFQ) developed by the Division of Human Nutrition of Wageningen University and Research, the Netherlands, was used to assess habitual intake of magnesium and calcium from the diet during the previous month. The magnesium or calcium content of a product was determined based on data from the Dutch food composition table of 2011 [20]. Dietary intake of magnesium and calcium was calculated for each food item based on frequency of intake, number of portions and portion size, as well as the type of product (e.g., whole grain bread or brown bread). The total amount of the nutrients consumed per day was calculated by adding all items containing the respective nutrient. The dietary intake of magnesium and calcium was adjusted for total energy intake using the residual method [21]. Dietary supplement use was assessed by a dietary supplement questionnaire developed by the Division of Human Nutrition of Wageningen University and Research [18]. Intake of magnesium and calcium from dietary supplements was not considered for the current study, due to the relatively low number of supplement users and the lack of details on exact dosages, frequency and compliance. However, we performed sensitivity analyses excluding patients who reported use of dietary supplements containing magnesium or calcium. The FFQs and dietary supplement questionnaires were filled out at diagnosis and six and 12 months after diagnosis. These three time points represent the usual dietary intake before surgery, during chemotherapy and six months after chemotherapy, respectively.

### 2.3. Chemotherapy-Induced Peripheral Neuropathy

Prevalence and severity of chronic CIPN were assessed using the ‘European Organization for Research and Treatment of Cancer Quality of Life Questionnaire to assess Chemotherapy Induced Peripheral Neuropathy’ (EORTC QLQ-CIPN16). This is an abbreviated version of the QLQ-CIPN20 [22], which is used in the COLON study. This questionnaire was filled out 12 months after diagnosis, representing approximately six months after the end of chemotherapy. The QLQ-CIPN16 consists of 16 questions of which eight assessing sensory symptoms and eight assessing motor symptoms. These questions were shown to be valid and reliable to assess patient-reported CIPN [22]. Patients reported the degree to which they have experienced sensory and motor symptoms during the past week using a 4-point Likert scale. Sensory and motor scale scores from the QLQ-CIPN16 ranged from 1–32. The scores of the QLQ-CIPN16 were linear transformed to a 0–100 scale. Based on mean scores of CIPN in the general Dutch population, the presence of CIPN was defined as >3.6 for the total CIPN16 score, >3.2 for the sensory sub-score and >3.8 for the motor sub-score [23]. Furthermore, we have evaluated severity of CIPN-related symptoms by specifically focusing on the CIPN scores, with higher scores indicating more severe CIPN. Missing values were handled by the mean-person imputation method [24], however, participants were excluded from analyses when more than two items in the QLQ-CIPN16 were missing.

### 2.4. Data Analysis

To study the association between magnesium and calcium and the prevalence of CIPN, prevalence ratios (PR) were calculated using a Cox proportional hazards regression model, with a fixed time variable. PRs were used, since odds ratios tend to overestimate the size of the association when the outcome is common [25]. Subsequently, among patients suffering from CIPN, the association between magnesium or calcium intake and the severity of CIPN was assessed by using multivariable linear regression analysis.

The Shapiro-Wilk test was used to determine whether data were normally distributed. Dietary intake of magnesium, calcium, vitamin D, total energy, as well as CIPN scores were natural-log transformed. To adjust for potential confounding, the models were adjusted for age, gender as well as energy adjusted dietary calcium or magnesium and vitamin D intake. When a variable changed the regression coefficient of the independent variable with 10% or more, the variable was considered to be a confounder and was added as a covariate to the model. Self-reported magnesium or calcium supplement use, diabetes mellitus, physical activity, smoking, as well as B vitamins and alcohol intake did not influence the observed associations. There was a strong correlation between protein and magnesium intake (*r* = 0.81), as well as between protein and calcium intake (*r* = 0.80), therefore we did not adjust for protein intake in the final model. Sensitivity analyses were performed for oxaliplatin-containing chemotherapy (OX), patients with colon cancer and non-supplement users. Stratified analysis was done for age (<65 and ≥65 years of age) to assess possible age differences.

Statistical analyses were performed in SAS 9.4 (SAS Institute, Cary, NC, USA). Ninety-five percent confidence intervals (95% CIs), not containing 1 for the Cox regression analyses and not containing 0 for the multivariable regression analyses, represent statistically significant associations [26].

## 3. Results

### 3.1. Patients Characteristics

In total, 196 CRC patients from 12 hospitals in the Netherlands were included in this study. The majority of the patients started with OX (*n* = 166, 85%), while 23 (11%) patients received capecitabine monotherapy (Table 1). In our study population, 160 patients (82%) reported CIPN 12 months after diagnosis. Among these patients, both sensory as well as motor CIPN symptoms were commonly reported (81% and 76%, respectively). Common sensory symptoms included tingling and numbness in fingers and toes, whereas difficulty with manipulating small objects and opening jars or bottles were commonly reported motor symptoms. The severity of sensory symptoms was higher than the severity of motor symptoms (mean score 20.8 and 12.5, respectively).

The median intake of magnesium was 317 mg/day and for calcium this was 851 g/day. The most important food sources of magnesium in our population were whole grain bread and nuts. Also, coffee, dairy products, dark chocolate, banana and legumes were common sources of magnesium. For calcium the most important food source was dairy products, especially cheese, yoghurt and milk. The intake of magnesium was below the estimated average requirement of 350 mg/day for men and 265 mg/day for women [27] in 48% of our study population at the time of diagnosis. During and after chemotherapy, 65% and 57% of the patients had an intake below the estimated average requirement, respectively. The intake of calcium was below the estimated average requirement of 800 mg/day for man <70 years and 1000 mg/day for man >70 years and 1000 mg for women [28] in 58% of our population at the time of diagnosis, 59% during treatment and 65% after treatment.

### 3.2. Dietary Magnesium or Calcium Intake and CIPN

Dietary intake of magnesium during chemotherapy was associated with the prevalence of chronic CIPN (PR 0.53, 95% CI 0.32, 0.90) (Table 2). Among patients suffering from CIPN, a higher dietary intake of magnesium was associated with less severe symptoms of CIPN (β −1.08, 95% CI −1.95, −0.22 for the intake during chemotherapy and β −0.93, 95% CI −1.81, −0.06 for the intake after chemotherapy) (Table 3). Dietary intake of calcium was not associated with the prevalence (Table 2) and severity (Table 3) of CIPN.

Sensitivity analyses including only patients receiving OX (*n* = 166) or only patients with colon cancer (*n* = 181) or only non-supplement users (*n* = 160–171) showed similar results (Table 4).

Stratified analyses for age showed statistically significant associations between magnesium intake at all three time points and the severity of CIPN for patients aged 65 and older (*n* = 88), but not for patients younger than 65 years (*n* = 108) (data not shown).

## 4. Discussion

The aim of this study was to assess the association between magnesium and calcium intake and CIPN in a prospective cohort of CRC patients. Dietary intake of magnesium during chemotherapy was associated with a lower prevalence of CIPN. A higher dietary intake of magnesium, but not calcium, during and after chemotherapy was associated with a lower severity of total CIPN symptoms. 

The prevalence of CIPN approximately six months after chemotherapy, i.e., 12 months after diagnosis, was higher than expected (81%) in our study population consisting of 196 CRC patients treated with adjuvant chemotherapy. Argyriou et al. reported a prevalence of 40% for CIPN in CRC patients six to eight months after finalizing their treatment containing OX [1]. CIPN is related to various risk factors, including treatment schedule, dose per course, cumulative dose, time of infusion and pre-existing peripheral neuropathy [1]. These factors likely vary between studies and countries. Furthermore, difference in the prevalence of CIPN may be explained by different methods to assess CIPN. Most previous studies used criteria of the National Cancer Institute (NCI-CTCAE) or the total neuropathy score (TNSc) to assess CIPN [4,5,6,7]. These methods are based on clinical examination, while the QLQ-CIPN16 is a patient-reported assessment of CIPN. The use of the QLQ-CIPN16 is inherent to the large-scale setting of the COLON cohort study. Recent studies compared several commonly used methods to assess CIPN [29,30]. A high correlation was found for the NCI-CTCAE and the EORTC QLQ-CIPN [29,30]. Furthermore, we did expect a high prevalence of sensory symptoms, and not motor symptoms, since OX is mainly associated with chronic sensory CIPN [1,31]. Although motor symptoms were also commonly reported in our study, it should be noted that the severity of motor symptoms was relatively low in the present study (mean score 12.5 versus 20.8 for sensory symptoms).

In the present study, we found that a higher magnesium intake was associated with a lower severity of chronic CIPN, whereas no association for calcium was found. Both magnesium and calcium are involved in electric excitability of neurons and muscle contraction [8]. A potential explanation for the association between magnesium and the severity of CIPN is the role of magnesium in the neuromuscular system and nervous tissue conduction [32]. It has been supposed that CIPN is caused by the stimulating effect of OX on neural excitability due to re-configuration of sodium channels in the cell membrane [33]. Magnesium (and calcium) are hypothesized to decrease OX-induced hyper-excitability [34,35], thereby limiting damage of neurons. In addition, magnesium specifically plays a role in membrane integrity and stability [32]. 

In the present study, we focused for the first time on dietary intake of magnesium and calcium in relation to CIPN. It should be noted that magnesium levels in blood are tightly regulated [36]. When circulating levels of magnesium are low, other tissues such as bone and muscle provide magnesium to restore circulating magnesium levels. With a low magnesium intake, body stores of magnesium could be depleted, while circulating levels are still in the healthy range [3,37]. Among our study population, 65% of the patients had an intake below the estimated average requirement of 350 mg/day for men and 265 mg/day for women [28] during chemotherapy. Hypothetically, especially patients with a low intake of magnesium could benefit from additional intake of magnesium, as a higher intake could restore depleted body stores of magnesium and thereby increases availability of magnesium in muscles and nerves. Stratified analyses for age showed a stronger association in patients aged 65 years and older. In this group the percentages of patients with a magnesium intake below the estimated average requirement was higher compared to participants younger than 65. In addition, during chemotherapy more participants had a magnesium intake below the estimated average requirement compared to before and after chemotherapy. These results indicate that an optimal magnesium status throughout treatment is important. Further studies are needed to confirm these findings and to determine the clinical relevance of the reported association between magnesium and CIPN.

The present study has some limitations. First, intake from dietary supplements was not considered because of the relative limited number of supplement users. In addition detailed data on dosage, frequency and compliance was lacking and supplement use was not consistent over the study duration. Also, the mineral content of the drinking water was not considered. Magnesium and calcium from drinking water contribute to the total magnesium and calcium intake. In the Netherlands, magnesium and calcium content in the tap water ranges from 1.7–26.2 mg/L (1–8% of the total intake) and 15–157 mg/L (1–17% of the total intake), respectively [38]. Second, although we explored the possible interaction between magnesium or calcium and many nutrients like B vitamins, calcium, vitamin D, vitamin E and alcohol, we could not exclude the possibility that other nutrients or bioactive compounds contributed to the observed effects. Third, the prevalence of pre-existing CIPN was not taken into account. Pre-existing CIPN is a risk factor for developing chronic CIPN [1]. Diabetes mellitus is an important cause of peripheral neuropathy [39]. However, in the present study, adjustment for self-reported diabetes mellitus did not influence the observed associations. In addition, we have not been able to take specific information on treatment-related factors, such as cumulative dose of chemotherapy and use of specific medications that may have influenced magnesium status into account. However, in the specific setting of studies focusing on long-term (chronic) toxicities, dose is a complicated factor. Patients who received a low (cumulative) dose may have experienced severe (acute) toxicities which have resulted in a dose reduction or premature discontinuation of therapy. Because of severe toxicities, among which potentially CIPN, an increased risk of chronic CIPN on the long-term may be expected. On the other hand, patients who completed their scheduled treatment, and hence received a high (cumulative) dose, may also have an increased risk of CIPN because of extensive exposure to the cytotoxic regimens. Furthermore, although information on clinical and socio-demographic characteristics of patients who did not fill out the QLQ-CIPN16 (*n* = 67, 23%) was available, it remains unknown why they did not fill out the questionnaires. It could be that these patients suffered from CIPN symptoms in their hands, resulting in selection which theoretically could decrease the validity of the present study. However, we do not expect that these non-responses had a major impact on the results of our study as the overall response rate was high (77%). The sample size of this study was relatively small (*n* = 196) and restricted to CRC patients and therefore we cannot state yet if generalization of our results to other cancer patients or chemotherapeutic agents is justified. Finally, we did not measure blood levels of magnesium and calcium. It should be noted, however, that the specific objective of this study was to investigate the association between dietary intake of magnesium and CIPN. Next to that, circulating levels are tightly regulated and not representative for magnesium levels in muscles and bone [37]. 

The present study also has important strengths. First of all, this is the first prospective cohort study which assessed the association between habitual dietary intake of magnesium or calcium and CIPN. Our data extend and complement existing evidence regarding the association between magnesium, calcium and neuropathy. Previous studies focusing on infusions with magnesium and calcium showed inconsistent findings and relied on acute exposure, while we assessed habitual, long-term intake of magnesium and calcium. In addition, previous studies focused on acute CIPN (during and directly after chemotherapy), while we focused on chronic and persistent CIPN. Therefore, this study provides an important contribution to the limited knowledge on chronic CIPN and its association with diet before, during and after chemotherapy. Furthermore, in the present study we used the 16 items of the QLQ-CIPN20 that are considered to be valid and reliable [22]. This approach resulted in a clinically relevant estimation of the prevalence and severity of CIPN compared to the QLQ-CIPN20 [22]. Due the availability of detailed data on diet and other clinical and lifestyle factors, we could adjust for the most plausible confounders, although residual confounding can never be fully excluded. 

## 5. Conclusions

The results of this study showed that a higher dietary magnesium intake was associated with a lower prevalence and less severe CIPN symptoms among CRC patients who received adjuvant chemotherapy. Further studies are needed to confirm our findings and to provide a solid basis for future recommendations directed towards the intake of magnesium before and during chemotherapy.

## Figures and Tables

**Table 1:** Characteristics of colorectal cancer patients who received adjuvant chemotherapy by prevalence of chemotherapy-induced peripheral chemotherapy (CIPN).

		CIPN	
	Total Population *n* = 196	CIPN No *n* = 36 (18%)	Yes ^1^ *n* = 160 (82%)
Gender, women	71 (36%)	12 (33%)	59 (37%)
Age (years)	64.0 (59.8–68.1)	65.4 (59.0–68.2)	63.7 (59.9–68.0)
Diabetes mellitus (yes) ^2^	15 (8%)	3 (8%)	12 (8%)
Physical activity (meeting norm) ^3^	143 (73%)	25 (69%)	118 (74%)
Tumor stage			
Stage II	15 (8%)	2 (6%)	13 (8%)
Stage III	145 (74%)	28 (76%)	117 (73%)
Stage IV	21 (10%)	3 (9%)	18 (11%)
Missing	15 (8%)	3 (9%)	12 (8%)
Cancer site			
Colon	181 (93%)	33 (92%)	148 (93%)
Rectum	14 (7%)	3 (8%)	11 (7%)
Missing	1 (0%)	0 (0%)	1 (0%)
Type of chemotherapy			
Oxaliplatin-containing (OX)	166 (85%)	27 (75%)	139 (87%)
Capecitabine monotherapy	23 (12%)	9 (25%)	14 (9%)
Other	2 (1%)	0 (0%)	2 (1%)
Missing	5 (2%)	0 (0%)	5 (3%)
Dietary factors			
Magnesium intake from diet (mg/day) ^2,4^	317 (261–383)	325 (261–396)	313 (261–380)
Use of magnesium supplements (yes)	36 (18%)	6 (17%)	30 (18%)
Calcium intake from diet (mg/day) ^2,4^	851 (621–1143)	789 (597–1155)	861 (631–1123)
Use of calcium supplements (yes)	35 (18%)	6 (17%)	29 (18%)
Vitamin D intake from diet (mg/day) ^2,4^	3.1 (2.4–4.1)	2.8 (2.2–3.7)	3.2 (2.4–4.2)
Total energy intake (kcal/day) ^2,4^	1893 (1534–2265)	1898 (1547–2230)	1893 (1534–2285)
CIPN ^5^			
Total score ^6^	14.6 (6.3–26.4)	0.0 (0–2.1)	16.7 (10.4–29.2)
Sensory score ^7^	16.7 (4.2–37.5)	0.0 (0–0)	20.8 (12.5–37.5)
Motor score ^8^	8.3 (4.2–20.8)	0.0 (0–0)	12.5 (8.3–20.8)

Values presented are median (quartile 1–quartile 3) or number (percentage). ^1^ Cut-off point for CIPN total score: 3.6, ^2^ Assessed at diagnosis. ^3^ Meeting the Dutch physical activity guideline of 150 min per week of moderate intensive exercise at baseline, ^4^ Intake is missing for two patients, ^5^ Assessed 12 months after diagnosis. ^6^ Data for one patient missing. ^7^ Data for two patients missing. ^8^ Data for five patients missing.

**Table 2:** Association between dietary magnesium or calcium intake and the prevalence of chronic chemotherapy-induced neuropathy in colorectal cancer patients receiving adjuvant chemotherapy.

	*n*/Events	Prevalence Ratio (95% CI) Total Score CIPN16 ^1^	*n*/Events	Sensory Symptoms ^1^	*n*/Events	Motor Symptoms ^1^
**Magnesium intake**						
**Crude model**						
At diagnosis	194/158	0.81 (0.51, 1.29)	194/157	0.98 (0.64, 1.52)	194/147	0.95 (0.54, 1.67)
During chemotherapy	192/156	**0.57 (0.35, 0.92)**	192/156	0.70 (0.44, 1.12)	192/145	0.59 (0.33, 1.05)
After chemotherapy	181/147	0.67 (0.43, 1.05)	181/146	0.86 (0.57, 1.31)	181/136	0.61 (0.36, 1.03)
**Full model**						
At diagnosis	194/158	0.69 (0.42, 1.13)	194/157	0.84 (0.51, 1.39)	194/147	0.81 (0.43, 1.52)
During chemotherapy	192/156	**0.53 (0.32, 0.90)**	192/156	0.67 (0.40, 1.10)	192/145	0.57 (0.30, 1.09)
After chemotherapy	181/147	0.61 (0.37, 1.03)	181/146	0.84 (0.51, 1.37)	181/136	**0.51 (0.28, 0.93)**
**Calcium intake**						
**Crude model**						
At diagnosis	194/158	1.15 (0.93, 1.41)	194/157	1.20 (0.98, 1.46)	194/147	1.12 (0.89, 1.41)
During chemotherapy	192/156	0.97 (0.83, 1.13)	192/156	1.02 (0.87, 1.19)	192/145	0.89 (0.73, 1.09)
After Chemotherapy	181/147	1.03 (0.86, 1.23)	181/146	1.08 (0.90, 1.30)	181/136	0.96 (0.78, 1.17)
**Full model**						
At diagnosis	194/158	1.21 (0.95, 1.56)	194/157	1.24 (0.97, 1.58)	194/147	1.15 (0.88, 1.50)
During chemotherapy	192/156	1.03 (0.86, 1.23)	192/156	1.06 (0.89, 1.28)	192/145	0.92 (0.73, 1.16)
After Chemotherapy	181/147	1.08 (0.89, 1.32)	181/146	1.10 (0.89, 1.35)	181/136	0.99 (0.79, 1.24)

Analyses were performed using a Cox proportional hazard regression model, with a fixed time variable. Crude models: energy-adjusted intake of magnesium or calcium. Full model for magnesium was adjusted for, energy-adjusted dietary calcium and vitamin D intake as well as age and gender. Full model for calcium was adjusted for, energy-adjusted dietary magnesium and vitamin D intake as well as age and gender. ^1^ CIPN was defined as a score >3.6 for the total CIPN16 score, >3.2 for the sensory sub-score and >3.8 for the motor sub-score.

**Table 3:** Associations between dietary magnesium or calcium intake and severity of chronic chemotherapy-induced neuropathy in colorectal cancer patients receiving adjuvant chemotherapy.

		Beta (95% CI)				
	*n*	Total Score CIPN16	*n*	Sensory Symptoms	*n*	Motor Symptoms
**Magnesium intake**						
**Crude model**						
At diagnosis	158	−0.68 (−1.45, 0.08)	157	**−1.09 (−1,90 −0.27)**	147	−0.45 (−1.33, 0.43)
During chemotherapy	156	**−1.21 (−2.01, −0.41)**	156	**−1.43 (−2.30, −0.57)**	145	**−1.25 (−2.16, −0.35)**
After chemotherapy	147	**−0.81 (−1.59, −0.04)**	146	**−1.05 (−1.87, −0.22)**	136	−0.64 (−1.48, 0.21)
**Full model**						
At diagnosis	158	−0.65 (−1,52, 0.20)	157	**−1.10 (−2.01, −0.18)**	147	−0.36 (−1.36, 0.63)
During chemotherapy	156	**−1.08 (−1.95, −0.22)**	156	**−1.24 (−2.17, −0.32)**	145	**−1.25 (−2.24, −0.27)**
After chemotherapy	147	**−0.93 (−1.81, −0.06)**	146	**−1.11 (−2.04, −0.19)**	136	−0.76 (−1.73, 0.21)
**Calcium intake**						
**Crude model**						
At diagnosis	158	−0.20 (−0.55, 0.14)	157	−0.31 (−0.68, 0.06)	147	−0.19 (−0.56, 0.18)
During chemotherapy	156	−0.31 (−0.62, 0.00)	156	−0.39 (−0.74, −0.04)	145	−0.31 (−0.66, 0.04)
After chemotherapy	147	−0.19 (−0.55, 0.17)	146	−0.36 (−0.75, 0.03)	136	−0.04 (−0.42, 0.35)
**Full model**						
At diagnosis	158	−0.05 (−0.45, 0.35)	157	0.03 (−0.40, 0.45)	147	−0.19 (−0.61, 0.23)
During chemotherapy	156	−0.16 (−0.51, 0.19)	156	−0.14 (−0.52, 0.23)	145	−0.19 (−0.57, 0.19)
After chemotherapy	147	−0.03 (−0.42, 0.35)	146	−0.10 (−0.52, 0.32)	136	0.01 (−0.40, 0.43)

Analyses were performed using multivariable linear regression analyses. Crude models: energy-adjusted intake of magnesium or calcium. Full model for magnesium was adjusted for energy-adjusted dietary calcium and vitamin D intake as well as age and gender. Full model for calcium was adjusted for energy-adjusted dietary magnesium and vitamin D intake as well as age and gender.

**Table 4:** Sensitivity analyses for the associations between dietary magnesium or calcium intake and severity of chronic chemotherapy-induced neuropathy.

		Beta (95% CI)				
	*n*	Total Score CIPN16	*n*	Sensory Symptoms	*n*	Motor Symptoms
**Patients receiving oxaliplatin-containing chemotherapy**						
**Magnesium**						
At diagnosis	137	−0.68 (−1.58, 0.22)	138	**−1.01 (−1.97, −0.05)**	126	−0.48 (−0.53, 0.57)
During chemotherapy	136	**−0.90 (−1.80, −0.00)**	137	**−1.10 (−2.06, −0.14)**	125	**−1.28 (−2.33, −0.24)**
After chemotherapy	132	**−0.94 (−1.85, −0.02)**	133	**−1.10 (−2.07, −0.12)**	121	−0.94 (−1.98, 0.10)
**Calcium**						
At diagnosis	137	0.00 (−0.42, 0.42)	138	0.08 (−0.36, 0.53)	132	−0.19 (−0.63, 0.26)
During chemotherapy	136	−0.18 (−0.54; 0.18)	137	−0.11 (−0.51; 0.28)	125	−0.28 (−0.69; 0.13)
After chemotherapy	132	0.00 (−0.39, 0.41)	133	−0.06 (−0.49, 0.38)	121	0.01 (−0.43, 0.45)
**Not supplement users**						
**Magnesium**						
At diagnosis	128	−0.80 (−1.80, 0.20)	126	−0.99 (−2.08, 0.10)	119	−0.68 (−1.83, 0.48)
During chemotherapy	136	**−1.24 (−2.21, −0.27)**	134	**−1.20 (−2.27, −0.13)**	128	**−1.81 (−2.90, −0.71)**
After chemotherapy	129	**−1.34 (−2.30, −0.37)**	126	**−1.29 (−2.37, −0.21)**	119	**−1.38 (−2.44, −0.32)**
**Calcium**						
At diagnosis	127	−0.06 (−0.50, 0.37)	127	0.06 (−0.41, 0.53)	119	−0.08 (−0.56, 0.38)
During chemotherapy	140	−0.12 (−0.48, 0.23)	138	−0.07 (−0.45, 0.32)	132	−0.20 (−0.60, 0.20)
After chemotherapy	127	−0.00 (−0.41, 0.42)	130	−0.09 (−0.54, 0.36)	119	0.09 (−0.35, 0.53)
**Colon cancer patients**						
**Magnesium**						
At diagnosis	146	−0.66 (−1.54, 0.22)	145	**−1.11 (−2.04, −0.18)**	135	−0.34 (−1.37, 0.68)
During chemotherapy	145	**−1.13 (−2.03, −0.23)**	145	**−1.34 (−2.30, −0.38)**	134	**−1.30 (−2.33, −0.26)**
After chemotherapy	139	−0.80 (−1.71, 0.11)	137	**−0.98 (−1.95, −0.02)**	128	−0.66 (−1.67, 0.36)
**Calcium**						
At diagnosis	146	−0.00 (−0.42, 0.41)	145	0.07 (−0.37, 0.51)	135	−0.15 (−0.58, 0.28)
During chemotherapy	145	−0.11 (−0.48, 0.25)	145	−0.07 (−0.47, 0.35)	134	−0.16 (−0.57, 0.26)
After chemotherapy	139	−0.03 (−0.45, 0.39)	137	−0.11 (−0.56, 0.34)	128	0.04 (−0.41, 0.50)

Analyses were performed using multivariable linear regression analyses. Models for magnesium was adjusted for energy-adjusted dietary calcium and vitamin D intake as well as age and gender. Models for calcium was adjusted for energy-adjusted dietary magnesium and vitamin D intake as well as age and gender.
